# Now or never: Organizational forgetting as a determinant of knowledge sharing and cross-boundary innovation

**DOI:** 10.3389/fpsyg.2022.1042990

**Published:** 2022-11-23

**Authors:** Xiaoyu Qu, Adnan Khan, Sajjad Ali

**Affiliations:** ^1^School of Management, Dalian Polytechnic University, Dalian, China; ^2^School of Management, Jiangsu University, Zhenjiang, China

**Keywords:** cross-boundary innovation, organizational forgetting, binary knowledge sharing, institutionalized organizational mission, exploitative knowledge sharing

## Abstract

This study constructs a theoretical model to test and prove that organizational forgetting influences cross-boundary innovation and testifies to the moderating role of Institutionalized organizational mission in the said relationship. Data was collected through a convenient sampling technique from 353 middle and senior managers of entrepreneurial enterprises in China through online and offline modes. Additionally, we used confirmatory factor analysis, multiple regression, and bootstrap analysis to verify hypotheses using Analysis of a moment structures and Statistical Package for the Social Sciences latest versions. The results show that organizational forgetting has a significantly positive impact on cross-boundary innovation and binary knowledge sharing plays a mediating role in the relationship between organizational forgetting and cross-boundary innovation. Moreover, the mediating effect of exploitative knowledge sharing on the relationship between organizational forgetting and cross-boundary innovation is more substantial than exploratory knowledge sharing. This study separates the impact mechanism of exploitative and exploratory knowledge sharing as a mediator unanimously and proves that Institutionalized organizational mission has a significant moderating role in the relationship between organizational forgetting and cross-boundary innovation. This research offers significant implications for Chinese enterprises to bolster cross-boundary innovation to achieve growth.

## Introduction

Creativity and innovation are the vital driving forces of modern firms and businesses (Acar et al., 2019). In the era of “Internet +,” Big data, IoT, and the sharing economy have opened the doors for enterprises to think beyond their traditional ways of innovation (Khan et al., 2022,c). New technologies have facilitated businesses and offered more challenges to cope with. Modern firms must be more agile in the current scenarios to adopt the latest technologies and respond to dynamic business environments (Harraf et al., 2015).

In a highly competitive and changing environment, organizational rigidity is not conducive to an enterprise’s success in the long run and could halt the chances of creativity and innovation (Soltwisch, 2015). Thus, most enterprises aim to bring cross-boundary innovation, offering several associated benefits (Kertcher et al., 2020). Thus the motivation behind the study could be illustrated as; cross-boundary innovation with tangible and intangible benefits involving industry, system, culture, etc., which can help enterprises eliminate core rigidity and strengthen their competitiveness (Levina and Vaast, 2005). Therefore, realizing cross-boundary innovation is becoming a critical problem for enterprises to solve. Cross-boundary innovation aims to extend organizational boundaries for new ideas.

This study attempts to answer the following research questions through the lens of the dynamic capability view, primarily focusing on “what are organizational boundaries, and under what conditions will occur across? How could cross-boundary innovation motivation help enterprises develop new ideas? Furthermore, how can the implementation mechanism of cross-boundary innovation be built in modern enterprises?

Knowledge management infrastructure capabilities lay a foundation for knowledge creation and accumulation in cross-boundary innovation of enterprises (Khan and Tao, 2022; Khan et al., 2022). Effective knowledge sharing is the standard cognition of enterprises to realize cross-boundary innovation, but the implementation effect is unsatisfactory (Edmondson and Harvey, 2018). Enterprises with exploitative knowledge sharing are limited to exchanging and transmitting actual knowledge, instead entrapped in knowledge rigidity. However, enterprises with too much exploratory knowledge sharing may emphasize acquiring and exploring new knowledge and taking excessive risks. The single knowledge-sharing method is not conducive to cross-boundary innovation; thus, binary knowledge-sharing has become an essential way for enterprises to realize cross-boundary innovation (Meissner et al., 2021). Therefore, it is necessary to systematically reveal the relationship between binary knowledge sharing and cross-boundary innovation.

This research study proposes the conceptual framework that binary knowledge sharing can trigger cross-boundary innovation, and institutionalized organizational mission plays a crucial role in achieving this goal. Knowledge sharing alone cannot improve the enterprise knowledge system (Akgün et al., 2014), as cross-boundary innovation also requires organizations to break inherent rules and conventions. Prahalad and Hamel (2009) also reflect that firms must not follow the conventions to realize cross-boundary innovation.

Organizational forgetting, as a capability of self-change and innovation (Kluge and Gronau, 2018), is necessary for knowledge sharing (Anser et al., 2020) and cross-boundary innovation. However, the existing literature rarely discusses combining the three, i.e., organizational forgetting, binary knowledge sharing, and cross-boundary innovation. Therefore, this study takes organizational forgetting as a base to further study the relationship between binary knowledge sharing and cross-boundary innovation. On the notions of innovation search theory (Nelson and Winter, 1977), the degree of organizational mission rigidities influences whether organizational forgetfulness fosters organizational innovation or not. An institutionalized organizational mission is essential, reducing organizational transformability and flexibility (McKinley et al., 2014). Besides, the risk of cross-boundary innovation in enterprises is relatively high, and there will be more apparent conflicts between institutionalized organizational missions and concurrent innovation goals. Therefore, this research study proposes institutionalized organizational mission as a moderating variable to examine the interaction between organizational forgetting and institutionalized organizational mission on cross-boundary innovation behavior.

To empirically justify the said relationships, we collected data from entrepreneurial firms in china. Entrepreneurial enterprises are good at identifying market gaps and can take risks and explore. These firms are increasingly keen to innovate to create value and diversity. China’s government and business environment encourage these firms to flourish to contribute to the country’s overall economic development. Owing to the popularity and innovative products, these firms’ researchers’ offered services and decided to choose them as their study’s sample. This research study uses multiple regression analysis and a structural equation model to verify the mediating role of binary knowledge sharing in the relationship between organizational forgetting and cross-boundary innovation and the moderating role of institutionalized organizational mission on the relationship between organizational forgetting and cross-boundary innovation.

### Dynamic capability view

Modern enterprises equip themselves with the needed capabilities and resources to stay in competition (Teece et al., 2009; Pisano, 2017). On the notion of DCV, our research formulates its propositions that enterprises have to forget the traditional approaches, techniques, and knowledge in order to achieve cross-boundary innovation.

## Hypothesis

### Organizational forgetting and cross-boundary innovation

Cross-boundary innovation urges enterprises to break through and cross the border from the original limits, either by collision, cross, or fusion in different areas, to form a new operation practice and innovation system to create value and competitive advantage (Zahra, 1993). The term “innovation” in cross-boundary innovation is not general; it could be transformative, a breakthrough, or leading innovation. The “crossover” in cross-boundary innovation can span the boundaries of cognition, behavior, time, space, culture, system, or industry. In contrast, organizational forgetting refers to how enterprises discard or change old norms and establish new ones to cope with the modern business environment (Tsang and Zahra, 2008). Organizational forgetting includes a change in thinking, beliefs, traditional knowledge, practices, etc. (Yang et al., 2014).

The continuous development and growth of the enterprises could be stagnant after a particular period, as it bases its decisions on inherent thinking and logic. As a strategic behavior of an enterprise, cross-boundary innovation at this phase can essentially be seen as a riskier practice that challenges the intrinsic thinking logic and behavior patterns (Amabile et al., 1996). Now the primary purpose of the enterprises should be to come out of the fixed patterns and cross the professional barriers, seeking and establishing connections between different boundaries. Being bound by inherent boundaries and dominated by traditional thinking could result in rigid concepts and behavior patterns that may not be favorable for cross-boundary innovation. Firms must accept new resources and capabilities outside organizational boundaries to improve innovation performance (Teece et al., 2016).

Therefore, the original thinking, beliefs, and conventions must be forgotten (Akgün et al., 2006). An enterprise needs new knowledge, information, and ideas for active forgetting to trigger innovation and change (Becker, 2010; Chi, 2021). Thus organizational forgetting ability could urge searching for new knowledge, ideas, and practices to achieve cross-boundary innovation. It could be done by integrating traditional knowledge and wisdom with up-to-date knowledge (Wong et al., 2015). Consequently, organizational forgetting would be an effective and essential mechanism for enterprises to realize cross-boundary innovation. Therefore following the basic assumption of DCV that firms have to upgrade themselves with the needed capabilities of modern times, we propose that:

*H1*: Organizational forgetting has a positive impact on cross-boundary innovation.

### Organizational forgetting and binary knowledge sharing

Knowledge sharing refers to the knowledge exchange process sensed by the knowledge transferor and the receiver for reciprocity. Im and Rai (2008) proposed two ways of knowledge sharing from organizational learning, i.e., exploitative knowledge sharing and exploratory knowledge sharing. Exploitative knowledge sharing emphasizes improvement, optimization, and efficiency and is the knowledge exchange of risk-averse activities. Exploratory knowledge sharing emphasizes searching, experimenting, and taking risks to exchange and create new knowledge and exchange to pursue risky activities.

The organizational inertia will make the enterprise form a rigid knowledge-sharing network and low efficiency of the learning path (Švarc and Dabić, 2021). Traditional wisdom halts the chances of the progress of modern enterprises to innovate. The static mode may make the enterprises path-dependent in learning and innovation, and path dependence on knowledge acquisition and sharing is negative (Meziaz et al., 2001). The outdated organizational inertia will negatively affect the organizational ability to identify, absorb, and transfer knowledge sharing between enterprises (Fowler et al., 2000).

Knowledge absorption capacity is an essential factor of successful knowledge sharing in enterprises. Organizational forgetting is subletting old knowledge, traditions, and norms to establish new knowledge. Additionally, organizational forgetting could urge enterprises to train and improve knowledge transfer abilities to enhance cross-boundary innovation. “Establishing the new” in organizational forgetting means the change of enterprises’ knowledge network structure, interaction, and sharing mechanism (Ojansivu et al., 2021).

As organizational forgetting occurs, the new knowledge-sharing networks will produce stronger links, which may reduce or even remove path dependence in knowledge transfer and its negative influence (Reagans and McEvily, 2003). It can make the enterprise more adaptable to the current market and industry trends, encouraging the exploration of new knowledge. In addition, forgetting useless, old, and repeated knowledge can free up more storage space for enterprises, promote them to exchange knowledge more frequently, and absorb more valuable and up-to-date knowledge (Cegarra-Navarro and Dewhurst, 2006; Ghasemi et al., 2021). From the DCV’s perspective, firms have to forget the traditional mechanism of doing business to achieve something novel. To sum up, the following hypotheses are proposed:

*H2*: Organizational forgetting has a positive effect on binary knowledge sharing.

*H2a*: Organizational forgetting has a positive effect on exploitative knowledge sharing.

*H2b*: Organizational forgetting has a positive impact on exploratory knowledge sharing.

### Binary knowledge sharing and cross-boundary innovation

The acquisition of new knowledge in different fields is the key to the success of cross-boundary innovation. However, enterprises will significantly reduce the adaptability and efficiency of cross-boundary innovation by blindly exploring new fields without paying attention to exchanging existing sound knowledge (Anser et al., 2020; Shahzad et al., 2020) within enterprises. Exploitative knowledge sharing can promote the implementation of cross-boundary innovation. First, enterprises will enhance the depth and breadth of employees’ cognition of the current knowledge structure through continuous exploitative knowledge sharing, which will help enterprises recognize and correctly choose the “boundary” suitable for them to realize cross-boundary innovation. The knowledge base view also explains that exploitative knowledge sharing deepens the existing knowledge base of enterprises and improves enterprises’ efficiency and ability to use this knowledge to provide enterprises with new perspectives, ideas, and approaches to solve problems (Berchicci, 2013; Shahzad et al., 2020; Ghasemi et al., 2021). Secondly, continuous communication and knowledge exchange in several specific fields of an enterprise is likely to break through the original knowledge boundary, make the enterprise not constrained by the familiar, and promote cross-boundary thinking and creativity (Caner and Tyler, 2015; Meissner et al., 2021). Exploratory knowledge sharing can also promote the implementation of cross-boundary innovation. First, understanding new fields and exploring new knowledge are essential ways enterprises implement cross-boundary innovation. The role of exploratory knowledge sharing is to promote the communication between enterprises and other organizations in different fields to help enterprises identify the new direction of future markets, search and obtain external information, and fill in the gaps of knowledge required by enterprises in cross-boundary fields (Muscio et al., 2017). When an enterprise forms a diversified knowledge system (Brix, 2017), it will inspire cross-boundary innovation and improve the matching degree of knowledge and opportunities. Secondly, the innovativeness and risk-taking of exploratory knowledge sharing (Camisón and Forés, 2010, 2011) can help enterprises go out of the routine and cross boundaries, carry out transformative and breakthrough innovation activities with creative thinking, and stimulate enterprises to create new product portfolios with competitive advantages. Once again expanding the domains of DCV, the following hypotheses are proposed:

*H3*: Binary knowledge sharing has a positive impact on cross-boundary innovation.

*H3a*: Exploitative knowledge sharing has a positive impact on cross-boundary innovation.

*H3b*: Exploratory knowledge sharing has a positive impact on cross-boundary innovation.

### The mediating role of binary knowledge sharing

Organizational forgetting of cross-boundary innovation is not a straightforward linear process. Organizational forgetting can indirectly affect cross-boundary innovation through binary knowledge sharing. Specifically, organizational forgetting promotes exchanging and transmitting existing valuable knowledge and experience (Huang et al., 2018). Organizational forgetting also improves learning efficiency, reduces the dependence on existing resources, and encourages exploring new knowledge and markets by enabling enterprises to look for alternate ways (Bongso et al., 2020).

Thus, the first impetus gained by enterprises is through organizational forgetting towards cross-boundary innovation. However, how this knowledge would be transferred to achieve cross-boundary innovation is primarily complex for enterprises (Pham et al., 2021). This research proposes that binary knowledge sharing could play its role in facilitating the modern firm to transfer this knowledge in such circumstances.

Additionally, Binary knowledge sharing can optimize the knowledge space of enterprises, which may enhance enterprises’ creativity and positively promote cross-boundary innovation. Thus, it can be concluded as; organizational forgetting stimulates cross-boundary creativity (Andersen and Kragh, 2015), reduces organizational rigidity and inertia, and simultaneously promotes cross-boundary innovation through binary knowledge sharing (Tweedt, 2018). Some scholars have also confirmed that the impact of organizational forgetting on innovation and performance is indirect. Wong et al. (2015) verified the mediating role of organizational learning in the relationship between organizational forgetting and organizational performance. Therefore, this study attempts to make further inferences based on DCV and proposes the following hypotheses:

*H4*: Binary knowledge sharing plays a mediating role in the relationship between organizational forgetting and cross-boundary innovation.

*H4a*: Exploitative knowledge sharing plays a mediating role in the relationship between organizational forgetting and cross-boundary innovation.

*H4b*: Exploratory knowledge sharing plays a mediating role in the relationship between organizational forgetting and cross-boundary innovation.

### The moderating role of institutionalized organizational mission

The Organizational mission reflects the value orientation, development direction, goals, positioning, and societal obligations (Coase, 1988). An institutionalized organizational mission can effectively guide various activities in organizational operations. A highly institutionalized organizational mission will be less flexible in various business activities, so it is difficult for enterprises to bear the adverse consequences of organizational forgetting and innovation. In contrast, a joint institutionalized organizational mission offers relatively low obstacles to implementing organizational forgetting and innovation strategies. An enterprise with a high institutional organization mission restricts employee behavior (Babu and Chalam, 2016), sticks to the original rules, and relies on experience to perform the routinized task. Thus, minimize the chances of stimulating crossover thinking and creative enthusiasm that is not favorable to cross-boundary innovation. In addition, the high institutional organizational mission molds managers’ and employees’ behavior to avoid risks and uncertainties.

On the other hand, enterprises with a joint institutionalized organization mission often have higher adaptability and are willing to seek new trends and ideas (Kopaneva, 2019). Consequently, improve business insights and the ability to foresee contemporary market trends. Such enterprises can constantly search for and grasp new opportunities, enhancing their cross-boundary innovation chances.

Therefore, the following hypotheses are proposed:

*H5*: Institutionalized organizational mission negatively moderates organizational forgetting and cross-boundary innovation.

**Figure 1:**
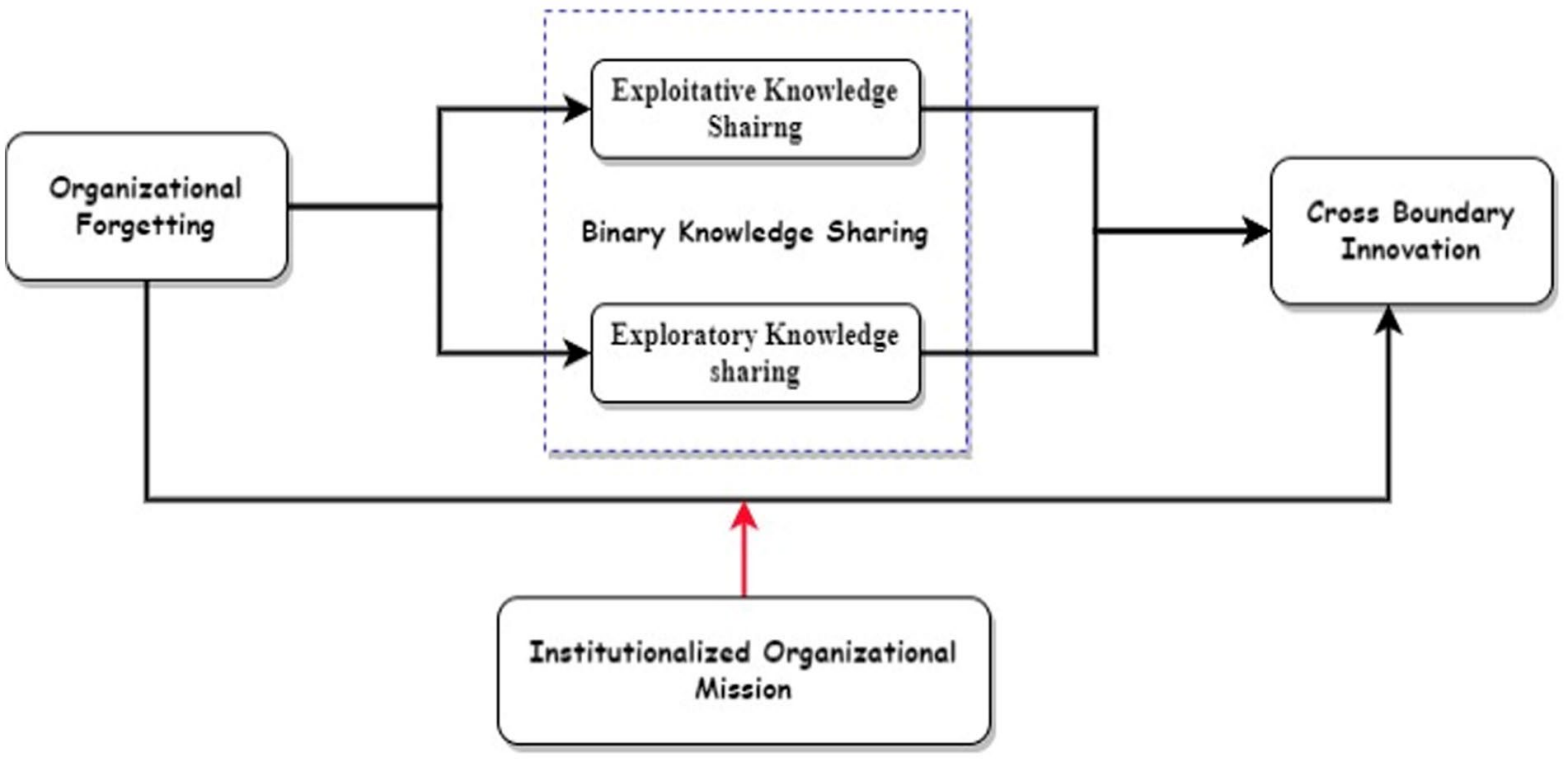
Research model.

The conceptual framework of this research is presented in Figure 1.

## Research methods

SEM is a widespread technique; it reflects the comprehensive mechanism of associated relationships among the variables. It determines the relationships on the outer and inner model fronts, leaving less error margin between the constructs (Hair et al., 2011, 2013). PLS-SEM is a more descriptive technique, and at the first stage, it facilitates the analysis of the measurement model and depicts the value to assure the validity and reliability of the data; recommended for organizational-level studies where the responses are taped from individuals (Chin, 1998; Hair et al., 2017). For these reasons, we used the SEM technique to analyze the data using the latest version of SPSS and AMOS.

### Research samples

With the continuous emergence of crossover, entrepreneurial enterprises show many specific cross-boundary innovation behaviors. Entrepreneurial enterprises refer to some relatively young enterprises that can expand their scale and increase profits (Bhidé, 2001; Safferstone, 2011). There is no strict restriction on the establishment period; they can be established for 1 year or more than 20 years (Zott and Amit, 2008). This research takes entrepreneurial enterprises as the research object using stratified sampling techniques. The selected samples include enterprises that have carried out cross-boundary innovation or participated in cross-boundary innovation activities in recent years. This research is about learning and innovation at the enterprise level. Therefore, middle and senior managers and members of the innovation team who are familiar with the overall situation of the enterprise are selected as respondents. This study adopts online and offline questionnaire surveys to obtain sample data. Relying mainly on the online mode questionnaire, got 209 responses, screen out 19 invalid questionnaires with all questions were responded with the same answer, appearing much extreme value of the items, and finally obtained 190 valid questionnaires. The offline method involved corporate visits and surveys of MBA students. Among them, 120 questionnaires were distributed and collected through visits to enterprises, and 108 were effectively collected. Sixty questionnaires were sent and collected through MBA students, and 55 of them were effectively collected.

Descriptive statistical information of 353 valid questionnaires obtained through online plus offline methods is as follows: 14.2, 31.4, 18.4, 22.1, and 13.9% of the enterprises have been working for 1–3 years, 4–6 years, 7–10 years, 11–15 years, and over 16 years. 18.7% of samples have 100 employees or less, 34.6% have 101–300 employees, and 46.7% have 301 employees or more. The firm is state-owned, accounting for 17.0%. Private accounting for 67.7%, foreign capital accounts for 15.3%, manufacturing accounts for 48.7%, service industry for 16.1%, Internet industry for 14.7%, and others for 20.5%. Due to the variety of data acquisition approaches and different acquisition times in this study, there was no common method biasness. For 190 valid questionnaires obtained online and 163 valid questionnaires obtained offline, a *t*-test of organizational forgetting, cross-boundary innovation, binary knowledge sharing, institutionalized organizational mission, and control variables (firm’s age, firm size, firm nature, and firm type) was conducted. The results show that the value of p of the F-test of organizational forgetting, cross-boundary innovation, binary knowledge sharing, and institutionalized organizational mission and control variables are higher than 0.05; the two batches of sample data of population variance have no significant difference. The double-tailed *p* values of the *t*-test are also higher than 0.05, indicating that no response deviation is not significant, and the two batches of data can be mixed. The demographics are presented in Table 1.

**Table 1:** Descriptive statistics (*n* = 353).

Sample characteristics	Classification standard	Percentage (%)
Firm’s age	1-3 year	14.2
	4–6 year	31.4
	7–10 year	18.4
	11–15 year	22.1
	16 years or more	13.9
Firm’s size	100 people or less	18.7
	101–300 people	34.6
	301 people or more	46.7
Firm’s nature	State-owned	17.0
	Private	67.7
	Foreign capital	15.3
Firm’s type	Manufacturing	48.7
	Service industry	16.1
	Internet industry	14.7
	Others	20.5

### Variables measurement

This study is based on the existing maturity scale adapted from previous proven research and used the Likert-7 subscales of each variable item (1 represents strongly disagree, 7 represents strongly agree). The details of all constructs and items are presented in Appendix 1.

The scale of organizational forgetting was designed by referring to the scale proposed by (Akgün et al., 2006), including ten items. The binary knowledge-sharing scale was designed by referring to the research (Im and Rai, 2008). The scale of exploratory knowledge sharing includes four items. The scale of the institutionalized organizational mission was designed by referring to (Mone et al., 1998), including four items. The scale of cross-boundary innovation was designed by referring to the innovation behavior scale (Zhou and George, 2001). According to the connotation and typical characteristics of cross-boundary innovation, the scale of cross-boundary innovation includes four items. The firm’s age, size, nature, and type are control variables.

## Data analysis and results

### Common method biasness and confirmatory factor analysis

In this study, the questionnaire was answered anonymously. At the same time, the questionnaire items were designed using the hidden meaning items. The variables or research intentions were not mentioned in the items to avoid incorrect answers from the respondents and effectively control the common method biases. Firstly, Haman’s single-factor detection method tested the degree of common method biases. The first principal component was 23.46% without rotation, indicating that the common method bias was acceptable. Then, by referring to the practice of (Podsakoff et al., 2003), the degree of common method biases is estimated through confirmatory factor analysis of the data. If the fitting degree of the one-factor model is optimal. As can be seen from Table 2, the one-factor model has the worst fitting degree (*χ*^2^/*df* = 16.269, RMSEA = 0.176, CFI = 0.649, NFI = 0.619, SRMR = 0.122). Therefore, there is no significant common method bias problem.

**Table 2:** Confirmatory factor analysis.

Model	*χ*^2^/*df*	RMSEA	CFI	NFI	SRMR
Five-factor model OF, EIKS, ERKS, OMS, CBI	2.331	0.078	0.926	0.905	0.049
Four-factor model OF, EIKS + ERKS, OMS, CBI	4.356	0.101	0.866	0.837	0.061
Three-factor model OF, EIKS + ERKS+OMS, CBI	8.656	0.129	0.800	0.768	0.082
Two-factor model OF + EIKS + ERKS + OMS, CBI	11.933	0.158	0.742	0.707	0.101
One factor model OF+EIKS + ERKS + OMS + CBI	16.269	0.176	0.649	0.619	0.122

OF, organizational forgetting. EIKS, exploitative knowledge sharing. ERKS, exploratory knowledge sharing. OMS, institutionalized organizational mission. CBI, cross-boundary innovation. + =two factors combine into one factor.

### Reliability and validity test

Exploratory factor analysis and confirmatory factor analysis are conducted on four variables: organizational forgetting, dual knowledge sharing, institutionalized organizational mission and cross-boundary innovation, respectively. According to the results of the exploratory factor analysis in Table 3, the factor loadings of the measurement items of each variable are all greater than 0.5, and the basic structure conforms to theoretical expectations. According to the confirmatory factor analysis results in Table 2, the fitting degree of the five-factor model (*χ*2/*df* = 2.331, RMSEA = 0.078, CFI = 0.926, NFI = 0.905, SRMR = 0.049) is the best, and each index reaches an acceptable value, indicating that the observation model is consistent with the data. The *t* value of the path coefficient between the measurement index and the latent variable of each main variable is greater than 2, indicating good convergent validity. Then, according to Table 3, the AVE values of organizational forgetting, dual knowledge sharing, institutionalized organizational mission and cross-boundary innovation are, respectively, 0.511, 0.546, 0.559, 0.595, and 0.577, all greater than 50%, and the square root of AVE value of each variable is greater than the correlation coefficient of its row and column in Table 4, indicating that it has good discrimination validity. In addition, Cronbach’s α coefficient is used to measure the reliability level of variables. According to Table 3, Cronbach’s *α* coefficient of four variables is all greater than 0.7, indicating that the scale has good reliability.

**Table 3:** Results of reliability and validity test.

Variables	Items	Cronbach’s *α*	KMO	Factor loadings	AVE
Organizational forgetting	OF1	0.832	0.820	0.733	0.511
	OF2			0.697	
	OF3			0.787	
	OF4			0.876	
	OF5			0.809	
	OF6			0.832	
	OF7			0.789	
	OF8			0.792	
	OF9			0.743	
	OF10			0.756	
Exploitative knowledge sharing	EIKS1	0.814	0.802	0.819	0.546
	EIKS2			0.788	
	EIKS3			0.738	
	EIKS4			0.722	
Exploratory knowledge sharing	ERKS1	0.828	0.812	0.746	0.559
	ERKS2			0.709	
	ERKS3			0.780	
	ERKS4			0.799	
Institutionalized organizational mission	OMS1	0.865	0.833	0.797	0.595
	OMS2			0.825	
	OMS3			0.774	
	OMS4			0.679	
Cross-boundary innovation	CBI1	0.839	0.829	0.727	0.577
	CBI2			0.822	
	CBI3			0.766	
	CBI4			0.882	

**Table 4:** Mean, standard deviation, and correlations of variables.

Variable	OF	EIKS	ERKS	OMS	CBI
OF	***0.715***				
EIKS	0.257^**^	***0.739***			
ERKS	0.566^***^	0.319^**^	***0.748***		
OMS	−0.265^**^	−0.194^*^	−0.325^**^	***0.771***	
CBI	0.445^***^	0.411^***^	0.498^***^	−0.396^***^	***0.760***
Mean	4.932	4.894	4.821	4.768	5.011
Std. Deviation	0.658	0.567	0.546	0.598	0.534

Diagonal bold italic numbers is square root of AVE; ^*^p < 0.05, ^**^p < 0.01, ^***^p < 0.001.

### Correlation analysis

The correlation analysis results among major variables are shown in Table 4. Organizational forgetting is significantly associated with cross-boundary innovation (*r* = 0.445, *p* < 0.001), exploitative knowledge sharing (*r* = 0.257, *p* < 0.01), exploratory knowledge sharing (*r* = 0.556, *p* < 0.001), and institutionalized organizational mission (*r* = −0.265, *p* < 0.01), exploitative knowledge sharing is significantly associated with cross-boundary innovation (*r* = 0.411, *p* < 0.001), exploratory knowledge sharing(*r* = 0.319, *p* < 0.01), and institutionalized organizational mission (*r* = −0.194, *p* < 0.05), exploratory knowledge sharing is significantly associated with cross-boundary innovation (*r* = 0.498, *p* < 0.001), and institutionalized organizational mission (*r* = −0.325, *p* < 0.01).

### Interpretation of empirical findings

#### Organizational forgetting and cross-boundary innovation

Multiple regression analysis was used to study the relationship between organizational forgetting and cross-boundary innovation. The results are shown in Table 5. The DW values of all models are around 2, and the VIF values of all models are below 5, *F* (*p*) < 0.05, indicating that the multi-collinearity problem is not severe. In Model 1, the standardized coefficient between organizational forgetting and cross-boundary innovation is 0.461 (*p* < 0.001), which indicates that organizational forgetting can significantly enhance cross-boundary innovation. Therefore, H1 is approved.

**Table 5:** Hierarchical regression analysis.

Variables	CBI	EIKS	ERKS	CBI	CBI	CBI	CBI	CBI
	Model 1	Model 2	Model 3	Model 4	Model 5	Model 6	Model 7	Model 8
Firm’s age	−0.012	−0.021	−0.018	−0.034	−0.026	−0.037	−0.019	−0.027
Firm’s size	0.019	0.011	0.012	0.023	0.015	0.035	0.033	0.028
Firm’s nature	0.003	−0.010	0.015	−0.009	0.020	0.024	0.002	−0.008
Firm’s type	0.009	−0.008	0.016	0.031	0.014	0.042	−0.004	0.013
OF	0.461***	0.232**	0.452***				0.290**	0.228**
EIKS				0.378***		0.273**		
ERKS					0.508***	0.356***		
OMS							−0.098	−0.126
OMS*OF								−0.311***
*R* ^2^	0.227	0.191	0. 265	0.293	0.358	0.412	0.287	0.398
*F*(*p*)	0.00							
DW	Around 2							
VIF	< 5							

^**^p < 0.01, ^***^p < 0.001.

#### Organizational forgetting and binary knowledge sharing

In the model of organizational forgetting and exploitative knowledge sharing (as shown in model 2, Table 5), the standardized coefficient of organizational forgetting and exploitative knowledge sharing is *β* = 0.232 (*p* < 0.01), explains the effect of organizational forgetting and exploitative knowledge sharing *R*^2^ = 0.191 (*p* < 0.01), which shows organizational forgetting has a significant positive influence on exploratory knowledge sharing. Therefore, H2a is approved. In the model of organizational forgetting and exploitative knowledge sharing (as shown in model 3 in Table 5), the standardized coefficient of organizational forgetting and exploratory knowledge sharing is *β* = 0.452 (*p* < 0.001), and the explains the effect of organizational forgetting and exploratory knowledge sharing is *R*^2^ = 0.265 (*p* < 0.01), which shows organizational forgetting has a significant positive influence on exploratory knowledge sharing. Therefore, H2b is approved.

#### Binary knowledge sharing and cross-boundary innovation

In the model of exploitative knowledge sharing and cross-boundary innovation (as shown in model 4 in Table 5), the standardized coefficient of exploitative knowledge sharing and cross-boundary innovation is *β* = 0.378 (*p* < 0.001). It explains the effect of exploitative knowledge sharing and cross-boundary innovation *R*^2^ = 0.293 (*p* < 0.01), which shows exploitative knowledge sharing significantly positively influences cross-boundary innovation. Therefore, H3a is verified. In the model of exploratory knowledge sharing and cross-boundary innovation (as shown in model 5 in Table 5), the standardized coefficient of exploratory knowledge sharing and cross-boundary innovation is *β* = 0.508 (*p* < 0.001). It explains the effect of exploratory knowledge sharing and cross-boundary innovation *R*^2^ = 0.358 (*p* < 0.01), which shows exploratory knowledge sharing significantly positively influences cross-boundary innovation. Therefore, H3b is verified. Exploitative and exploratory knowledge sharing was included in the regression model related to cross-boundary innovation (as shown in model 6 in Table 5). The standardized coefficient of exploitative knowledge sharing and cross-boundary innovation is *β* = 0.273 (*p* < 0.01), and the standardized coefficient of exploratory knowledge sharing and cross-boundary innovation is *β* = 0.356 (*p* < 0.001), which explains the effect of model 6 where *R*^2^ = 0.412 (*p* < 0.01). This further validates H3a and H3b.

#### The mediating role of binary knowledge sharing

Hayes (2013) proposed the mediating effect technique by installing a processing plug-in in SPSS; the bootstrap method was used in this study to verify further the multiple mediating effects of exploitative knowledge sharing and exploratory knowledge sharing on the relationship between organizational forgetting and cross-boundary innovation. The sample size of 5,000 was selected, and the 95% confidence interval was set. The results are shown in Table 6. The total effect of organizational forgetting on cross-boundary innovation is 0.377 (*p* < 0.05), and the 95% confidence interval is [0.215, 0.435], without 0, indicating that organizational forgetting has a significant effect on cross-boundary innovation. H1 is further verified. The total indirect effect of organizational forgetting on cross-boundary innovation is 0.202 (*p* < 0.05), and 95% confidence interval is [0.108, 0.404], without 0. The indirect effect coefficients of exploitative knowledge sharing and exploratory knowledge sharing are 0.063 (*p* < 0. 05) and 0.139 (*p* < 0. 05), and the 95% confidence intervals are, respectively, [0.017,0.102] and [0.054,0.205], both without 0, indicating that both exploitative knowledge sharing and exploratory knowledge sharing have significant mediating effects on the process of organizational forgetting affecting cross-boundary innovation. Then, the multivariate delta method was used to compare the mediating effect of exploitative knowledge sharing and exploratory knowledge sharing in organizational forgetting affecting cross-boundary innovation. The results showed that the difference between the two was 0.076 (p < 0. 05), 95% confidence interval is [0.029, 0.128]. It shows that the mediating effect of exploratory knowledge sharing is significantly stronger than exploitative knowledge sharing in organizational forgetting affecting cross-boundary innovation. The results indicate that organizational forgetting can influence cross-boundary innovation by promoting exploitative and exploratory knowledge sharing. Therefore, H4 is verified.

**Table 6:** Bootstrap analysis for significance test of mediation effect.

Mediation model	Effect	SE	95% confidence intervals	
			Lower limit	Upper limit
Total effect (*c* + *c*′): OF → CBI	0.377^*^	0.050	0.215	0.435
Direct effect(*c*′): OF → CBI	0.175^*^	0.057	0.033	0.219
Total indirect effect(c)	0.202^*^	0.046	0.108	0.404
Path 1(a_1_b_1_): OF → EIKS → CBI	0.063^*^	0.014	0.017	0.102
Path 2(a_2_b_2_): OF → ERKS → CBI	0.139^*^	0.035	0.054	0.205
DM: Path 2–Path 1	0.076^*^	0.018	0.029	0.128

^*^p < 0.05.

**Figure 2:**
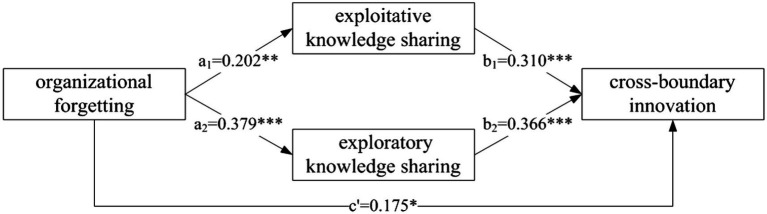
Path diagram of mediation effect of binary knowledge sharing.

To sum up, exploitative and exploratory knowledge sharing play multiple mediating roles between organizational forgetting and cross-boundary innovation, and their path of action is shown in Figure 2.

#### The moderating role of institutionalized organizational mission

In this study, multiple regression analysis examined the moderating role of institutionalized organizational mission in the direct impact of organizational forgetting on cross-boundary innovation. According to model 8 in Table 5, *R*^2^ rose to 0.398 from 0.227 in model 1, which shows that model 8 has a more substantial explanatory power. *F*-value is significant, standardized coefficient of institutionalized organizational mission * organizational forgetting is *β* = −0.311 (*p* < 0.001). The results indicate that institutionalized organizational mission negatively moderates the relationship between organizational forgetting and cross-boundary innovation. Therefore, H5 is verified.

## Discussion and implications

This study confirmed the relationship between organizational forgetting and cross-boundary innovation. It included the mediating role of binary knowledge sharing and the moderating role of institutionalized organizational mission in the conceptual model between organizational forgetting and cross-boundary innovation to explore the construction path of cross-boundary innovation further. The results show that:

1. Organizational forgetting positively affects cross-boundary innovation, indicating that the correct perception and implementation of organizational forgetting can help enterprises break existing rules, overcome core rigidity, and seek innovation opportunities through visible and invisible boundaries. Thus our study confirms the findings of (Bongso et al., 2020), who also states that organizational forgetting is a crucial element for innovations.
2. Binary knowledge sharing is the mediating variable in the relationship between organizational forgetting and cross-boundary innovation. In other words, organizational forgetting impacts cross-boundary innovation through exploitative and exploratory knowledge sharing. The mediating effect of exploratory knowledge sharing is more robust than exploitative knowledge sharing. This shows that the “abandon the old” and “discipline the new” in organizational forgetting can continuously promote enterprises. It provides storage space for new knowledge and promotes enterprise exploitative knowledge sharing and exploratory knowledge sharing. Knowledge transfer can ceaselessly result in creativity and smooth implementation of cross-boundary innovation. This study confirms the findings of binary knowledge sharing evident in the work of (Khan and Tao, 2022; Khan et al., 2022).
3. Institutionalized organizational mission has a negative moderating effect on the relationship between organizational forgetting and cross-boundary innovation; institutionalized organizational mission can weaken the positive effect of organizational forgetting on cross-boundary innovation. Furthermore, the institutional organizational mission weakens the recognition of organizational forgetting. Ojansivu et al. (2021) also concluded that firms’ outcomes might vary under the magnitude of institutionalized organizational mission. It inhibits the willingness and motivation of enterprises to break conventions and norms to carry out cross-boundary innovation through organizational forgetting. It may also reduce the possibility of enterprises grasping cross-boundary innovation opportunities and stimulate cross-boundary innovation thinking through organizational forgetting.

### Theoretical implications

The conceptual model of this study is based on the renowned theory of DCV that modern enterprises have to upgrade their skills, resources, and competencies for improved organizational outcomes. Thus, our study authenticates the notions of DCV by putting forward the view that, for cross-boundary innovation, enterprises must learn new skills and capabilities.

This study reveals the realization path of cross-boundary innovation. The theoretical and qualitative research in this domain mostly carried out case studies, explored the concept, characteristics, motivation, operational processes, etc., of cross-boundary innovation, and less explored its antecedent variable as a realization path of cross-boundary innovation. This study takes binary knowledge sharing as the mediating variable and institutionalized organizational mission as the moderating variable and constructs the effect path of organizational forgetting on cross-boundary innovation. The scope of discussion on the antecedent variables of cross-boundary innovation is enriched through empirical analysis, and the black box of the realization path of cross-boundary innovation is opened.

This study uses empirical research to test the acute effects of organizational forgetting and binary knowledge sharing on cross-boundary innovation. Few studies have discussed the influence of organizational forgetting and knowledge sharing on cross-boundary innovation, ignoring the strong effect of organizational forgetting and knowledge sharing on cross-boundary innovation. This study finds that organizational forgetting is essential for managers to make strategic changes and implement cross-boundary innovation. Organizational forgetting can bring new learning to the organization, bring deeper motivation for organizational change, and accelerate innovation and change compared with organizational memory. Therefore, organizational forgetting has a higher learning value and will have a more substantial incentive in driving cross-boundary innovation. In addition, this study expands the research perspective from a dual perspective, i.e., emphasizing the mediation role of binary knowledge sharing. The dual nature of knowledge sharing explores the mediating effects of exploitative and exploratory knowledge sharing on the relationship between organizational forgetting and cross-boundary innovation. It founds that organizational forgetting could affect cross-boundary innovation through exploitative knowledge sharing and exploratory knowledge sharing. It expanded the research perspectives and fields of mediating mechanisms of binary knowledge sharing.

This study finds that an institutionalized organizational mission is essential in cross-boundary innovation. Existing literature research indicates that the central role of the institutionalized organizational mission is to increase the organization’s legitimacy (Singh et al., 1986); this study introduces institutionalized organizational mission, which is an enterprise characteristic variable as a boundary condition to the concept model of cross-boundary innovation. However, it found that institutionalized organizational mission weakens the positive effect of organizational forgetting on cross-boundary innovation. The institutionalized organizational mission will rigid an organization and strengthen the path dependence of organization development. It becomes difficult for enterprises to have correct cognition and willingness to organizational forgetting. Such enterprises may find it hard to break through the original rules to implement cross-boundary innovation. This has important implications for organizational traits and forgetting synergistically influence cross-boundary innovation.

### Managerial implications

This research study has specific managerial implications for achieving cross-boundary innovation. First, Enterprises should attach importance to organizational forgetting management. Enterprise leaders may formulate corresponding rules and regulations and incentive measures to improve the enforcement of organizational forgetting and create an open atmosphere to optimize staff cognitive and behavioral patterns, stimulate creative thinking, and discard worthless old knowledge in time to improve the enterprise’s absorb knowledge effectively promote cross-boundary innovation. Secondly, enterprises should cultivate the dual mode of knowledge sharing. Enterprises should set up the concept of organizational ambidexterity inside and outside the organization. It could be done through case discussions, exchange of experience regularly, create a binary knowledge-sharing environment.

Furthermore, managers can train employees to be familiar with the enterprise’s existing work methods and knowledge structures and the dynamic change of the external environment. Managers should also update their employees about their industry’s development trends, emerging technologies, etc., to promote the internal and external value and cutting-edge knowledge. Finally, enterprises need to update their organizational mission regularly. In cross-boundary innovation, organizational forgetting must be combined with a smaller organizational mission that may play a synergistic role. Therefore, enterprise leaders need to recognize and appreciate the effect of a joint institutionalized organizational mission to encourage cross-boundary innovation. Compatibility of the organizational mission, organizational forgetting, and cross-boundary innovation can reduce the adverse effects of institutional organization missions.

## Limitations and future research

Although this study has reached some conclusions with absolute theoretical and practical value, some deficiencies need to be further expanded in subsequent studies due to the complexity of the research problem. This paper explores the relationship between organizational forgetting and cross-boundary innovation and examines the mediating and moderating effect of binary knowledge sharing and institutionalized organizational mission. In addition, there may be other mediating and moderating variables in the relationship between organizational forgetting and cross-boundary innovation, such as market opportunities, technological innovation, risk preferences, and cross-boundary experiences of decision-makers or management teams’ life-cycle characteristics of an enterprise. Future studies may further explore the mediating and moderating mechanism of other related variables on the relationship between organizational forgetting and cross-boundary innovation.

Secondly, this paper empirically examines the correlation between organizational forgetting, binary knowledge sharing, institutionalized organizational mission, and cross-boundary innovation. It fails to identify the multiple concurrent causal relationships among these variables. Based on the configuration perspective, future research may explore the configuration effect of organizational forgetting, binary knowledge sharing, institutionalized organizational mission, etc.

Finally, because the cross-section design is adopted in this paper, the research conclusion may differ from a longitudinal study or experimental study. Future research needs to consider applying multiple research methods to study the validity of the theoretical model more comprehensively. In addition, although some significant conclusions have been drawn in this paper, they are limited by the sample design, the mediating effect model, and the moderating effect model; the validity of the theoretical model in this paper needs to be further verified by more scientific and practical methods based on optimizing the sample selection, enlarging the sample size and perfecting the selection of control variables.

## Data availability statement

The raw data supporting the conclusions of this article will be made available by the authors, without undue reservation.

## Author contributions

All authors listed have made a substantial, direct, and intellectual contribution to the work and approved it for publication.

## Funding

This research work is funded by National Natural Science Foundation of China, Grant number 71802035.

## Conflict of interest

The authors declare that the research was conducted in the absence of any commercial or financial relationships that could be construed as a potential conflict of interest.

## Publisher’s note

All claims expressed in this article are solely those of the authors and do not necessarily represent those of their affiliated organizations, or those of the publisher, the editors and the reviewers. Any product that may be evaluated in this article, or claim that may be made by its manufacturer, is not guaranteed or endorsed by the publisher.

## Appendix 1

Variables measurement.

**Table:**

Variables	Items	Source
Organizational forgetting	OF1. The firm develops practical training and learning plans according to its own development needs	Akgün et al. (2006)
	OF2. The firm introduces management tools and develops new management processes according to its own development needs	
	OF3. The application of new technologies between different professions often requires technical exchange	
	OF4. The firm often encourages everyone to discuss the shortcomings of working methods and find a better way	
	OF5. New technologies are usually learned and digested before they are applied	
	OF6. The top executives often hold discussions to adjust the management mode	
	OF7. The firm often reviews corporate work processes and points out inadequacies	
	OF8. The firm often criticizes outdated ways of working	
	OF9. The firm periodically disposes of or destroys knowledge that has lost value	
	OF10. The firm often encourages employees to monitor each other and reminds them not to fall into the wrong old ways of working	
Exploitative knowledge sharing	EIKS1. Transfer and absorb new knowledge in existing fields	Im and Rai (2008)
	EIKS2. Exchange ideas and solutions frequently when problems arise in the development of new products/services	
	EIKS3. Exchange information and experience on improving existing evaluating methods of short-term performance objectives	
	EIKS4. Share working methods conducive to improving work efficiency	
Exploratory knowledge sharing	ERKS1. Transfer and absorb knowledge in new areas	
	ERKS2. Transfer and absorb the knowledge that helps expand the scope of the business	
	ERKS3. Transfer and absorb knowledge and information to open new markets	
	ERKS4. Exchange information and experience on achieving long-term strategic goals	
Institutionalized organizational mission	OMS1. The organizational mission is difficult to change due to environmental changes	Mone et al. (1998)
	OMS2. Organizational mission guides the choice of enterprise innovation strategy for a long time	
	OMS3. Organizational mission is not timely with environmental changes	
	OMS4. Organizational mission ensures the legitimacy of enterprise operation	
Cross-boundary innovation	CBI1. The firm often introduces elements from different fields to improve the operating conditions of related businesses	Zhou and George (2001)
	CBI2. The firm often explores opportunities and activities for innovation and development in different fields	
	CBI3. The firm often searches for new technologies, processes, technologies and/or product ideas	
	CBI4. The firm often comes up with practical ideas to improve performance	
